# Improving Depressive Symptoms of Post-stroke Depression Using the Shugan Jieyu Capsule: A Resting-State Functional Magnetic Resonance Imaging Study

**DOI:** 10.3389/fneur.2022.860290

**Published:** 2022-04-14

**Authors:** Guanqun Yao, Xiaoqian Zhang, Jing Li, Sha Liu, Xinrong Li, Pozi Liu, Yong Xu

**Affiliations:** ^1^School of Clinical Medicine, Tsinghua University, Beijing, China; ^2^Department of Psychiatry, Tsinghua University Yuquan Hospital, Beijing, China; ^3^Department of Psychiatry, First Hospital/First Clinical Medical College of Shanxi Medical University, Taiyuan, China; ^4^Shanxi Key Laboratory of Artificial Intelligence Assisted Diagnosis and Treatment for Mental Disorder, First Hospital of Shanxi Medical University, Taiyuan, China

**Keywords:** post-stroke depression, depressive symptoms, Shugan Jieyu capsule, functional magnetic resonance imaging, regional homogeneity, fractional amplitude of low-frequency fluctuation

## Abstract

Regional homogeneity (ReHo) and fractional amplitude of low-frequency fluctuation (fALFF) were used to detect the neuroimaging mechanism of Shugan Jieyu Capsule (SG) in ameliorating depression of post-stroke depression (PSD) patients. Fifteen PSD patients took SG for 8 weeks, completed the 24-item Hamilton Depression Scale (HAMD) assessment at the baseline and 8 weeks later, and underwent functional magnetic resonance imaging (fMRI) scanning. Twenty-one healthy controls (HCs) underwent these assessments at the baseline. We found that SG improved depression of PSD patients, in which ReHo values decreased in the left calcarine sulcus (CAL.L) and increased in the left superior frontal gyrus (SFG.L) of PSD patients at the baseline. The fALFF values of the left inferior parietal cortex (IPL.L) decreased in PSD patients at the baseline. Abnormal functional activities in the brain regions were reversed to normal levels after the administration of SG for 8 weeks. Receiver operating characteristic (ROC) analysis found that the changes in three altered brain regions could be used to differentiate PSD patients at the baseline and HCs. Average signal values of altered regions were related to depression in all subjects at the baseline. Our results suggest that SG may ameliorate depression of PSD patients by affecting brain region activity and local synchronization.

## Introduction

Post-stroke depression (PSD) mainly manifests as depression, loss of interest, loss of appetite, sleep disturbance, pessimistic sense of worthlessness, and even suicidal tendencies (1). PSD not only affects neurological repair of stroke patients and leads to severe cognitive impairment, but also affects rehabilitation efficacy after stroke and increases mortality and the recurrence of stroke (2). Compared to other diseases with a similar degree of disability, stroke patients are likely to exhibit depressive symptoms, suggesting that the etiology of PSD has a more complex neurobiological basis (3). However, the pathogenesis of PSD is still not clear. Recent researches on PSD mainly involved neurobiological mechanisms and social psychological mechanisms, such as immunization activities, education level and social support level (4). Additionally, one meta-analysis showed that PSD was not significantly related to the location of stroke lesions (5). Investigating the mechanisms of PSD and identifying effective treatment methods has become a topic of prolific investigation in clinical and basic research.

Due to the unique physical condition of PSD patients, conventional antidepressants may result in more side effects in PSD patients, and the effect of ameliorating depression of PSD patients is not ideal (6). In recent years, there have been increasing clinical attempts to use traditional Chinese medicine to treat PSD patients. Shugan Jieyu Capsule (SG), a traditional Chinese drug compound made from *Hypericum perforatum* and Acanthopanax, have been widely approved for the treatment of depression in China since 2008 (7). *Hypericum perforatum* effectively inhibits the reabsorption of neurotransmitters such as norepinephrine, 5-hydroxytryptamine, and dopamine, thus counteracting the effects of depression (8, 9). Acanthopanax senticosus suppresses resorption of central serotonin and increases concentration of monoamine transmitters in the synaptic cleft to achieve antidepressant effects (10). Furthermore, Acanthopanax senticosus regulates the body's endocrine system and central nervous system, and reduces oxidative stress damage (11). Collectively, the combination of *Hypericum perforatum* and Acanthopanax senticosus has a beneficial effect on improving depression. Additionally, previous studies have shown that for patients with mild to moderate depression, SG has a more effective safety profile and fewer side effects compared to selective serotonin reuptake inhibitors (SSRIs) (12). A large number of studies have shown that SG may ameliorate depressive symptoms of PSD patients, and shows better compliance and fewer side effects in PSD patients (13). However, the neurobiological mechanisms of this process are still unclear.

Resting state functional magnetic resonance (fMRI) detects the changes in blood oxygen and blood flow in the brain, and indirectly reflects neural activity by measuring the relationship between blood flow, blood oxygen, and oxygen consumption (14). Previous studies have found that depressive symptoms of PSD are related to the decrease in the gray matter volume, the reduction in anisotropy in the reward system, and the increase in free extracellular fluid (15). A longitudinal pharmacological study of depression has found that SG may improve depressive symptoms and cognitive function in depressed patients through functional regulation of the right caudate nucleus and the left orbitofrontal cortex (7). In addition, functional integration studies have shown that executive dysfunction in PSD patients is associated with the change in the internal functional connection of the resting state network, the functional over-connection between the default mode and the cognitive control network, and the reduction of the cross-hemispheric frontal and parietal functional connections (16).

In previous studies, the dynamic changes in the brain have been used to explore the possible mechanism of SG in ameliorating cognition of PSD patients (17). However, we didn't find a correlation between brain dynamics and depression improvement in PSD patients. Brain dynamics is a good indicator that has been proposed in recent years to explore changes in functional brain activity without directly comparing differences in spontaneous brain activity. Regional homogeneity (ReHo) and fractional amplitude of low-frequency fluctuation (fALFF) are classic indicators of spontaneous functional activity. Previous studies have shown that ReHo and fALFF may be associated with depressive symptoms in patients with depression (18, 19), which may help to find brain regions associated with improved depressive symptoms in PSD patients.

ReHo analysis shows that the voxels of a certain functional brain region have high consistency under certain conditions. By calculating the consistency between the time series of each voxel and its neighboring voxel, the Kendall's harmony coefficient (KCC) is obtained, which reflects the degree of synchronization of local brain neurons (20). Furthermore, the ReHo indicator may be used to detect abnormalities in the inherent local synchronization of the brain. A study on schizophrenia has found extensive abnormal ReHo values in the precuneus, inferior parietal lobule and other areas in the early stages of schizophrenia (21). A similar study in patients with depression has found that the ReHo values of the left precuneus and left lingual gyrus in depression patients are related to depression and cognition (22). Additionally, while ALFF describes intensity of brain activity in the local region between the voxels, it also has defects. Due to the signal noise generated during the scanning process, there will be a lot of energy in the ventricular position, and this high energy in the ventricular position does not necessarily have physiological significance. Furthermore, fALFF divides the energy of the calculated low-frequency signal by the power of the entire frequency band, which improves the sensitivity and specificity of signal detection (23). Previous studies have found that fALFF values of the right precuneus are negatively correlated with the number of depressive episodes in depressive patients (24). Therefore, ReHo and fALFF may be effective indicators to explore the neurobiological mechanisms of SG improving depressive symptoms in PSD patients.

In this study, we hypothesize that SG may ameliorate depressive symptoms in PSD patients and that this improvement would be related to brain region activity and local synchronization. The 24-item Hamilton Depression Scale (HAMD) was used to evaluate depression, and the combination of ReHo and fALFF was used to explore the neurobiological mechanisms of SG in ameliorating depression of PSD patients.

## Methods and Materials

### Subjects

Professional physicians selected suitable PSD patients for enrollment according to strict standards and recruited corresponding healthy individuals as controls. The inclusion criteria for the PSD patients were as follows: have a diagnosis of hemorrhagic or ischemic stroke; meet the DSM-5 diagnostic criteria for PSD, where depressive symptoms occur within 1 week to 3 months after the stroke; mild to moderate depression, HAMD scores between 8 and 24; Han nationality, right-handed, aged between 50 and 70 years old; meet the conditions of MRI scan (without any contraindications); are not allowed to use electroconvulsive therapy, psychotherapy, other types of psychiatric drugs or mood stabilizers within 8 weeks of medication.

The exclusion criteria were as follows: have severe physical obstacles, speech impediments, and other obstacles, unable to complete psychological measurement; have other serious physical diseases; have used or are using other antipsychotic drugs; are receiving therapies such as psychotherapy and physical therapy; have other mental illnesses or severe depression (HAMD > 24).

The recruited healthy volunteers (HCs) had no history of brain trauma or mental illness, could complete psychological tests, and met the conditions of magnetic resonance scanning without any contraindications. Fifteen PSD patients and 21 HCs were ultimately recruited. This study was approved by the Ethics Committee of Shanxi Medical University and was enrolled in the Chinese Clinical Trial Registry (clinical registration number: ChiCTR1900026358). All participants provided informed consent. The subjects and clinical data of this study were consistent with our previous study (17).

The patients took 1.44 g per day in two divided doses according to the instructions of SG. Professional psychological testers performed HAMD on PSD patients in the quiet assessment room during the baseline period and the 8th weekend of treatment (25). Professional imaging technicians performed brain MRI scans on PSD patients during the baseline period and the 8th weekend. The administration of SG occurred after the MRI scan at the baseline. After the administration of SG for 8 weeks, the MRI scan was performed immediately without a washout period. The HC group only performed HAMD and brain MRI scans at the baseline.

### Data Acquisition

Imaging data of all subjects were scanned by the imaging physicians in the MRI room using a 3T German Siemens scanner. The 3D T1 parameters were as follows: 160 transverse slices without gaps; voxel size = 0.9 × 0.9 × 1.2 mm^3^; repetition time/echo time (TR/TE) = 2,300/2.95 ms; matrix = 240 × 256; flip angle = 9°; Functional arguments were listed: voxel size = 3.75 × 3.75 × 4 mm^3^; flip angle = 90°; 32 transverse slices without gaps; 212 time points; TR/TE = 2,500/30 ms.

### Lesion Mapping

**Figure 1:**
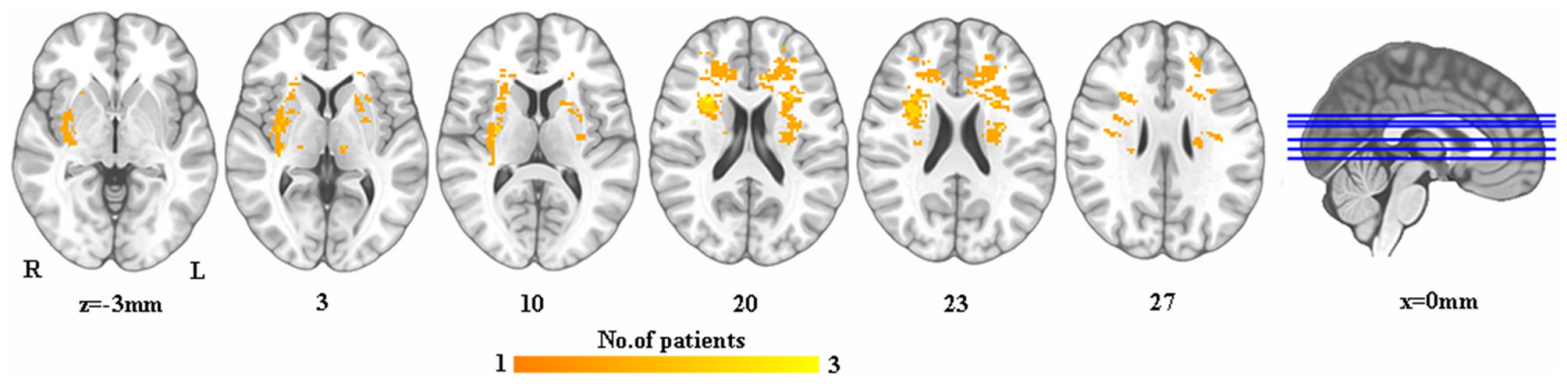
The lesion map in PSD patients. Warm colors indicate the overlay of PSD patient's lesions. PSD, post-stroke depression; L, left; R, right.

A lesion overlay was created for all PSD patients. A neurosurgeon manually marked the 3D T1 lesion contour images of each patient with MRIcron software (http://www.mccauslandcenter.sc.edu/mricro/mricron/). After spatial standardization of SPM12 toolkit, overlapping images of group lesions were constructed by combining individual lesion templates to form a unified group lesion template (Figure 1).

### Data Preprocessing

DPARSF (http://www.restfmri.net) and SPM12 toolkits (http://www.fil.ion.ucl.ac.uk/spm) were used to complete the preprocessing of functional data. The first 10 images of all subjects were deleted due to signal instability and the subjects' adaptation to scanning noise. The remaining images were corrected in time and space, and the translation/rotation measurement parameters of the functional image data were removed over ±2.5 mm/±2.5°. The individual average frame displacement (FD) was calculated based on the translation and rotation indices of the head movement parameters. FD exceeding 0.5 mm was regarded as an abnormal movement value and was used to eliminate the influence of head movement. The personal 3D T1 image was registered to the functional image, and the 3D T1 image was segmented and normalized to Montreal space (MNI) using a 12-parameter non-linear affine transformation. A cost correction script was used to eliminate lesion areas to avoid deviations caused by spatial standardization, that is, to exclude the influence of the lesion area signal on subsequent analysis (26). The functional image was resampled to 2 × 2 × 2 mm^3^ voxel size after spatial normalization. Then a Gaussian kernel of full-width at half-maximum of 6 mm was used for spatial smoothing. Furthermore, band pass filter (0.01–0.08 Hz) was used to correct the linear drift of the time point. Additionally, white matter signals, 24 movement parameters, and cerebrospinal fluid were eliminated as covariates.

### ReHo Analysis

The functional images required for ReHo analysis were not smoothed during preprocessing. For the time series similarity of functional areas, the KCC was used to measure the local consistency, and 27 adjacent voxels were defined as a functional area. The DPARSF software was utilized to calculate local consistency between the time series of each voxel and the time series of adjacent 26 voxels one by one to obtain the individual ReHo graph. Finally, Fisher-z transformation was used in ReHo values to improve data normality, and the resulting z-valued ReHo was used for subsequent statistical analysis.

### fALFF Analysis

To improve specificity and sensitivity, the fALFF value was calculated as the ratio of the power of a specific frequency band (0.01–0.08 Hz) to the power of the entire detection frequency band (0.01–0.25 Hz) for inhibiting non-specific signals in the functional imaging. Finally, Fisher-z transformation was used in fALFF values to improve data normality, and the resulting z-valued fALFF was used for subsequent statistical analysis.

### Statistical Analysis

The DPARSF toolkit was used to perform independent-sample *t*-tests on the ReHo and fALFF values of PSD patients before treatment (SG0W) and HCs, respectively. The Gaussian random field (GRF) correction was used for multiple comparison correction (voxel level *p* < 0.01 and cluster level *p* < 0.05). The average values of altered brain regions were extracted for *post hoc* comparison. Pearson correlation was performed to explore whether ReHo and fALFF values in altered brain regions were associated with HAMD scores in all subjects at the baseline (Bonferroni correction at *p* < 0.05). Furthermore, receiver operating characteristic (ROC) analysis was performed to determine optimal threshold for distinguishing between SG0W and HCs. Paired *t*-test was performed to compare SG0W and PSD patients after 8 weeks of treatment (SG8W), which analyzed the changes in the ReHo and fALFF values of the altered brain regions after the administration of SG for 8 weeks. In addition, the independent-sample *t*-test was performed to explore distinction of the scale scores between SG0W and HCs, and the paired *t*-test was performed to explore distinction of scale scores between SG0W and SG8W.

## Results

### Demographic and Clinical Features

There was no difference in sex (χ^2^ = 0.385, *p* = 0.535), age (Mann–Whitney *U* test, *p* = 0.181) and education level (Mann–Whitney *U* test, *p* = 0.241) between PSD patients and HCs, and the average volume of lesions in PSD patients was 1.65 ± 1.46 cm^3^ (Table 1). Furthermore, the HAMD scores of PSD patients were higher than that of HCs (*t* = 8.103, *p* < 0.001) at the baseline, and the HAMD score of SG8W were lower than that of SG0W (*t* = 3.911, *p* = 0.002).

**Table 1:** Demographic and clinical characteristics.

**Variable**	**SG0W**	**SG8W**	**HC**	***t*** **/χ^2^**	***p***
	**(***n*** = 15)**	**(***n*** = 15)**	**(***n*** = 21)**		
	**Mean ±SD**	**Mean ±SD**	**Mean ±SD**		
Age (years)	64.13 ± 6.01		60.67 ± 6.95	/	0.181^a^
Sex (male/female)	8/7		9/12	0.385	0.535^b^
Education (years)	9.40 ± 3.38		10.29 ± 2.61	/	0.241^a^
Duration (days)	62.87 ± 15.73				
HAMD	14.00 ± 6.02		2.95 ± 1.50	8.103	0.001^c^
	14.00 ± 6.02	6.73 ± 5.41		3.911	0.002^d^
**Stroke type**					
Ischemia	9 (60.0)				
Hemorrhage	6 (40.0)				
Lesion volume (cm^3^)	1.65 ± 1.46				
**Location of lesion**					
Basal ganglia	9 (60.0)				
Frontal lobe	3 (20.0)				
Thalamus	3 (20.0)				

*SG0W, post-stroke depressive patients before treatment; SG8W, post-stroke depressive patients after 8 weeks treatment; HCs, healthy controls*.

a *Mann–Whitney U test*.

b *Chi-squared test*.

c *Independent-sample t-test*.

d *Paired t-test*.

*HAMD, 24-item Hamilton Depression Scale*.

### ReHo Differences

**Figure 2:**
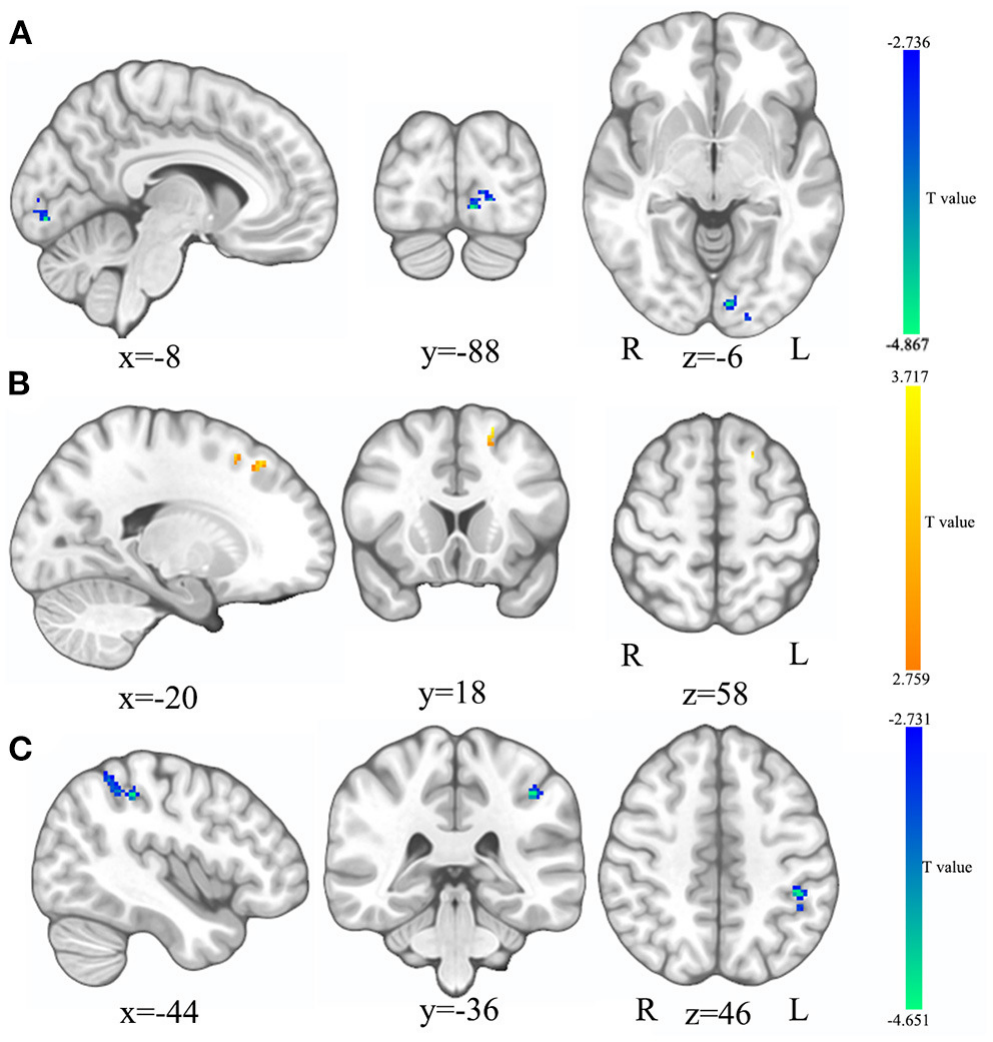
The comparison in ReHo and fALFF between HC and SG0W. **(A)** The brain region where the ReHo values of SG0W decreased; **(B)** The brain region where the ReHo values of SG0W increased; **(C)** The brain region where the fALFF values of SG0W decreased; SG0W, post-stroke depressive patients before treatment; HC, healthy controls; ReHo, regional homogeneity; fALFF, fractional amplitude of low-frequency fluctuation; L, left; R, right.

**Figure 3:**
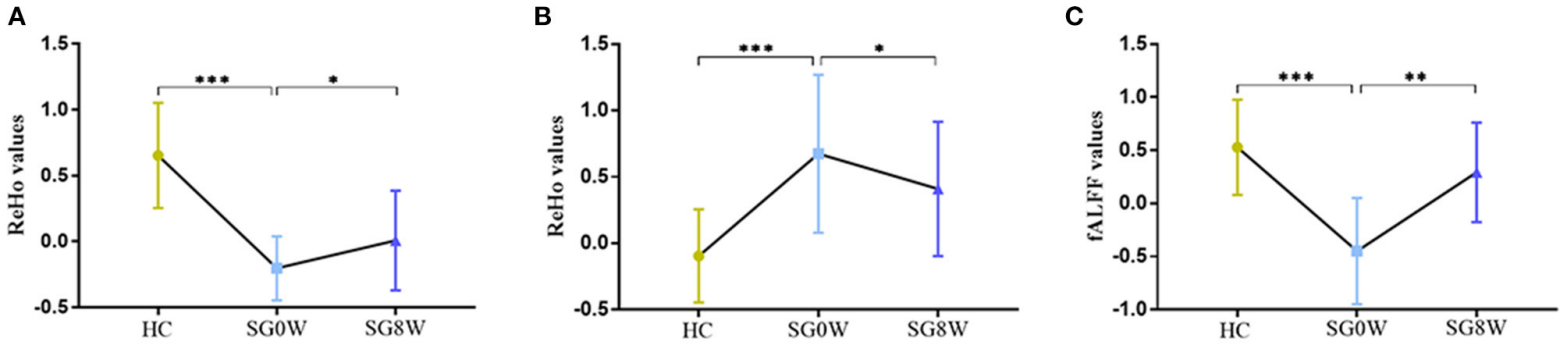
The *post hoc* comparison of the average signals in altered brain regions. **(A)** Comparison of ReHo values in the CAL.L; **(B)** Comparison of ReHo values in the SFG.L; **(C)** Comparison of fALFF values in the IPL.L; *p < 0.05, **p < 0.01, ***p < 0.001; SG0W, post-stroke depressive patients before treatment; SG8W, post-stroke depressive patients after 8 weeks treatment; HC, healthy controls; ReHo, regional homogeneity; fALFF, fractional amplitude of low-frequency fluctuation; CAL.L, left calcarine sulcus; SFG.L, left superior frontal gyrus; IPL.L, left inferior parietal cortex.

Compared to HCs, the ReHo values of the left calcarine sulcus (CAL.L) were lower, and the ReHo values of the left superior frontal gyrus (SFG.L) were higher (*p* < 0.05 GRF correction, cluster size >50 voxels) in PSD patients at the baseline (Figure 2 and Table 2). Average signals of altered brain regions were extracted for the *post hoc* comparison (Figure 3). The ReHo values of the CAL.L in PSD patients were lower than those in HCs at the baseline and increased after the administration of SG for 8 weeks. The ReHo values of the SFG.L in PSD patients were higher than those in HCs at the baseline and decreased after the administration of SG for 8 weeks.

**Table 2:** ReHo and fALFF differences between SG0W and HC.

**Brain areas**	**BA**	**MNI coordinates**			**Peak voxels**	***t***
		**X**	**y**	**z**		
**ReHo**						
SG0W < HC						
CAL.L	BA18	−8	−88	−6	65	−4.867
SG0W > HC
SFG.L	BA8	−20	18	58	55	3.717
**fALFF**						
SG0W < HC						
IPL.L	BA40	−44	−36	46	140	−4.651

*SG0W, post-stroke depressive patients before treatment; HC, healthy controls; ReHo, regional homogeneity; fALFF, fractional amplitude of low-frequency fluctuation; MNI, Montreal Neurological Institute; BA, Brodmann; CAL.L, left calcarine sulcus; SFG.L, left superior frontal gyrus; IPL.L, left inferior parietal cortex*.

### fALFF Differences

Compared to HCs, the fALFF values of left inferior parietal cortex (IPL.L) were lower in PSD patients at the baseline (*p* < 0.05 GRF correction, cluster size >50 voxels) (Figure 2 and Table 2). Average signals of altered brain regions were extracted for the *post hoc* comparison (Figure 3). Compared to HCs, the fALFF values of IPL.L in PSD patients were lower at the baseline, and increased after the administration of SG for 8 weeks.

### ROC Analysis

**Figure 4:**
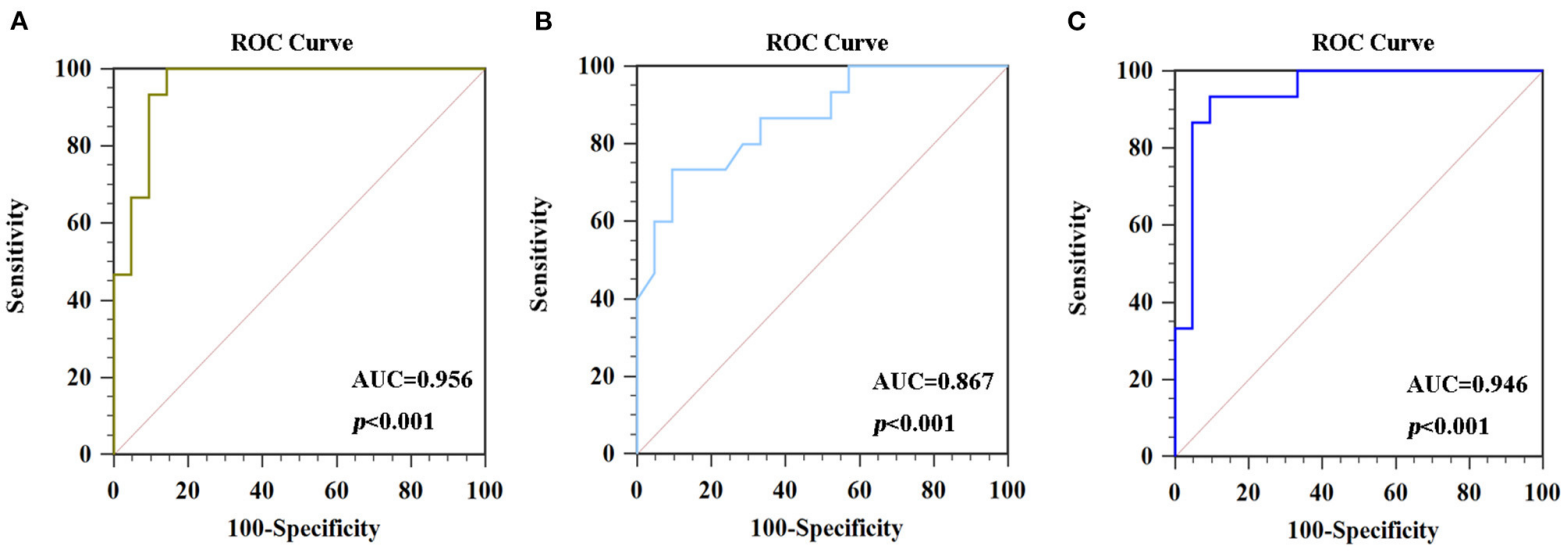
ROC curve analysis of average signals in altered brain regions. **(A)** The AUC was 0.956 (*p* < 0.001; 95% CI 0.829–0.996) for ReHo values in the CAL.L. **(B)** The AUC was 0.867 (*p* < 0.001; 95% CI 0.712–0.957) for ReHo values in the SFG.L. **(C)** The AUC was 0.946 (*p* < 0.001; 95% CI 0.816–0.994) for fALFF values in the IPL.L. ROC, receiver operating characteristic curves; AUC, area under the curve; ReHo, regional homogeneity; fALFF, fractional amplitude of low-frequency fluctuation.

The ROC curve was used to explore average ReHo values and fALFF values of altered brain regions, and the area under the ROC curve (AUC) was used to detect rate of diagnosis. The ROC analyse showed that the changes in three altered brain regions could be used to identify PSD patients and HCs: the ReHo values of CAL.L (0.956, *p* < 0.001), the ReHo values of the SFG.L (0.867, *p* < 0.001), and the fALFF values of IPL.L (0.946, *p* < 0.001) (Figure 4).

### Correlation Analysis

**Figure 5:**
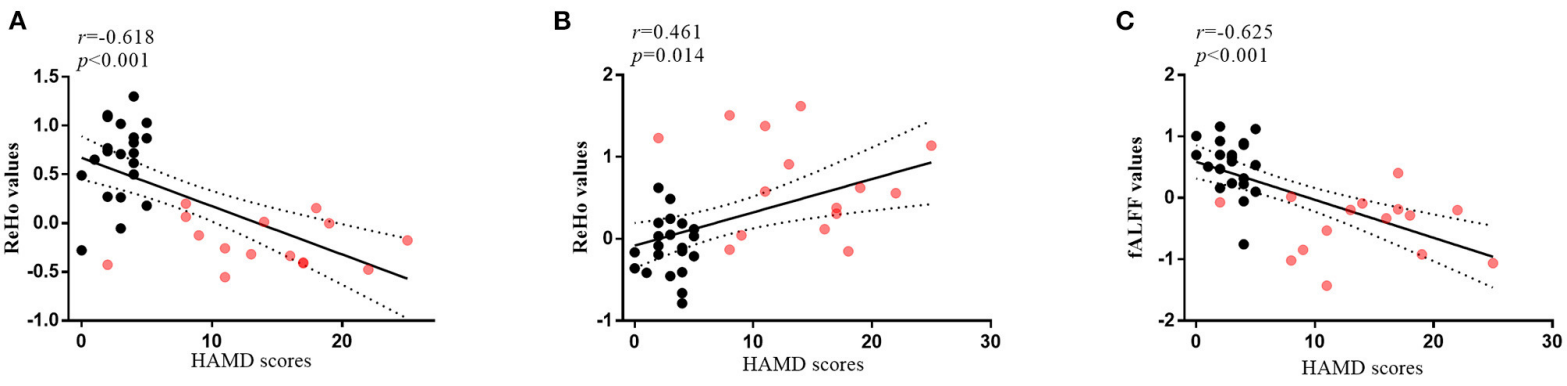
The correlations between altered brain regions and clinical features. **(A)** The correlation between HAMD scores and ReHo values of the CAL.L (*r* = −0.618, *p* < 0.001) in all subjects at the baseline; **(B)** The correlation between HAMD scores and ReHo values of the SFG.L (*r* = 0.461, *p* = 0.014) in all subjects at the baseline; **(C)** The correlation between HAMD scores and fALFF values of the IPL.L (*r* = −0.625, *p* < 0.001) in all subjects at the baseline; the red dots, patients with post-stroke depression at the baseline; the black dots, healthy controls; HAMD, 24-item Hamilton Depression Scale; ReHo, regional homogeneity; fALFF, fractional amplitude of low-frequency fluctuation; CAL.L, left calcarine sulcus; SFG.L, left superior frontal gyrus; IPL.L, left inferior parietal cortex.

As shown in Figure 5, the ReHo values of CAL.L and the fALFF values of IPL.L were negatively correlated with HAMD scores in all subjects at the baseline. The ReHo values of the SFG.L were positively correlated with HAMD scores in all subjects at the baseline.

## Discussion

In our previous study, we have found that dynamic low-frequency amplitude (dALFF) in the right precuneus and dynamic functional activity (dFC) in the right precuneus and left angular gyrus are reversed after drug administration and are correlated with cognitive function. However, our approach with ReHo and fALFF made some new discoveries. In this study, we detected that the depression symptoms of PSD patients were reduced after the administration of SG for 8 weeks, which was consistent with previous studies (17, 27). Additionally, the study found that ReHo values of CAL.L, SFG.L, and fALFF values of IPL.L in PSD patients were abnormal, which were reversed to normal values after the administration of SG for 8 weeks. Moreover, in the baseline period, the average signal values of altered brain areas were related to depressive symptoms, and ROC analysis showed that the three altered brain area changes could be used to distinguish PSD patients from HCs. Our findings may help understand the neuroimaging mechanisms of SG in improving depression of PSD patients.

In previous studies, it has been found that the functional connectivity of the default mode network (DMN) and the salient network (SN) in PSD patients is related to severity of depressive symptoms (28). Studies have also found that compared to stroke patients without depressive symptoms, PSD patients show abnormal functional connections between the left inferior gyrus and left inferior frontal gyrus, and this is related to the severity of depression (29). It has been further found that gray matter volume of the prefrontal and motor cortex in PSD patients is reduced, and the functional connectivity between it, anterior cingulate cortex, and the insula is abnormal (30). Additionally, depressive symptoms of PSD patients have been shown to be associated with lower functional connectivity between the left dorsolateral prefrontal lobe and the right superior gyrus (31). While previous studies have mostly explored the relationship between depressive symptoms and brain function integration in PSD patients based on functional connectivity and brain structure indicators, ours was the first to combine the ReHo and fALFF to explore both the relationship between depression and local brain activity in PSD patients. By understanding functional abnormalities of local brain activity involved in depressive symptoms of PSD patients, we may provide more effective and targeted interventions.

Previous studies have found that the CAL.L, an important area for visual and auditory processing, is involved in multiple independent networks and is involved in multisensory processing in visual, auditory, language, and emotional aspects (32). A study examining aphasia after stroke has shown that the activation of the CAL.L as a visual and auditory pathway is closely related to the processing of language and memory (33). Further studies have found that the ReHo abnormalities in the CAL.L and the left middle occipital gyrus are related to emotional facial recognition in patients with bipolar disorder, as well as interpersonal relationships in the social environment (34). We speculate that the dysfunction of the CAL.L may not only lead to impairments in emotional and social functioning, but also in language and memory in PSD patients. SG may directly enhance emotion and memory functions of PSD patients by affecting the local brain activity in this area.

The SFG is the center node of executive control network and emotion network, and abnormal function of SFG may induce depression (35). Dysfunction of the SFG may be associated with impaired emotional regulation and damage top-down control of the limbic region in depressive patients (36). One study has found that volume of gray matter in the SFG.L decrease in patients with depression, and that the functional connection between the SFG.L and the left hippocampus also decrease in conjunction with depressive symptoms (37). Further studies have found that the function of the SFG.L in depressive patients is abnormal compared to those with bipolar disorder or healthy controls, and that it may be related to the severity of rumination (38). It has been further found that the level of stress perception in adolescents is strongly correlated with spontaneous brain activity of the SFG.L, and that perceived stress mediates the relationship between the spontaneous activity of the SFG and depressive symptoms (39). In our current study, we observed that PSD patients showed a compensatory increase in spontaneous activity of the SFG.L in response to depressive symptoms, which tended to return to normal levels after SG administration for 8 weeks.

The IPL.L is associated with cognitive functions such as language recognition, memory processing, and attention processing (40, 41). Previous studies have shown that neural activity in the parietal cortex of primates is related to the relative subjective desire to act in strategic games and foraging, and that the parietal cortex is associated with the processing of rewards and tasks (42). There is also evidence to suggest that the IPL.L is related to cognitive control, directly linking cognitive control and motivational function in the brain (43). Furthermore, depressive symptoms of PSD patients have been shown to be associated with abnormal functional connectivity in the IPL.L in comparison to stroke patients without depressive symptoms (44), which is partially consistent with our findings. Additionally, the IPL.L is a crucial region in the DMN (45, 46). Abnormal DMN function in PSD patients has been observed to be related to the severity of depressive symptoms and impairment of cognitive function (47). The altered ReHo values of the IPL.L in our study may be associated with abnormal local brain activity and DMN dysfunction of PSD patients, and SG may improve depressive symptoms by reversing this abnormality.

However, this study also has some limitations. First, the sample size was small. PSD patients were generally older and had poor physical conditions due to stroke, and it was difficult to insist on long scanning periods of MRI scanning. PSD patients also had poor compliance, and the 8-week longitudinal follow-up increased the difficulty of collecting subjects. Future data should attempt to include more observations from a larger sample. Second, PSD patients showed heterogeneous stroke locations, and future studies should attempt to collect data from PSD patients with similar stroke locations. Finally, due to limited funds, we were only able to obtain baseline data from the control group. Time effect is also an important factor, and we will collect the control group data after 8 weeks in future studies.

In summary, our study shows that SG might ameliorate depression in PSD patients by affecting local brain activity and local synchronization. Our findings provide new insights for exploring the neurobiological mechanisms of SG in improving depression of PSD patients.

## Data Availability Statement

The raw data supporting the conclusions of this article will be made available by the authors, without undue reservation.

## Ethics Statement

The studies involving human participants were reviewed and approved by the Ethics Committee of Shanxi Medical University. The patients/participants provided their written informed consent to participate in this study.

## Author Contributions

YX and PL designed and funded the study. GY completed data analysis and article writing. XZ and JL revised the article. SL and XL completed the MRI scan and scale evaluation of the subjects. All authors contributed to the article and approved the submitted version.

## Funding

This study was funded by the National Natural Science Foundation of China (81971601 and 81571319).

## Conflict of Interest

The authors declare that the research was conducted in the absence of any commercial or financial relationships that could be construed as a potential conflict of interest.

## Publisher's Note

All claims expressed in this article are solely those of the authors and do not necessarily represent those of their affiliated organizations, or those of the publisher, the editors and the reviewers. Any product that may be evaluated in this article, or claim that may be made by its manufacturer, is not guaranteed or endorsed by the publisher.

## References

[1] Robinson RG Jorge RE. Post-stroke depression: a review. Am J Psychiatry. (2016) 173:221–31. 10.1176/appi.ajp.2015.15030363 26684921

[2] Loubinoux I Kronenberg G Endres M Schumann-Bard P Freret T Filipkowski RK. Post-stroke depression: mechanisms, translation and therapy. J Cell Mol Med. (2012) 16:1961–9. 10.1111/j.1582-4934.2012.01555.x 22348642 PMC3822966

[3] Das J G KR. Post stroke depression: the sequelae of cerebral stroke. Neurosci Biobehav Rev. (2018) 90:104–14. 10.1016/j.neubiorev.2018.04.005 29656030

[4] Wang Z Shi Y Liu F Jia N Gao J Pang X. Diversiform etiologies for post-stroke depression. Front Psychiatry. (2018) 9:761. 10.3389/fpsyt.2018.00761 30728786 PMC6351464

[5] Wei N Yong W Li X Zhou Y Deng M Zhu H. Post-stroke depression and lesion location: a systematic review. J Neurol. (2015) 262:81–90. 10.1007/s00415-014-7534-1 25308633

[6] Legg LA Tilney R Hsieh CF Wu S Lundström E Rudberg AS. Selective serotonin reuptake inhibitors (SSRIs) for stroke recovery. Cochrane Database Syst Rev. (2019) 2019:CD009286. 10.1002/14651858.CD009286.pub3 31769878 PMC6953348

[7] Liu S Zhao W Li Y Li X Li J Cao H. Improve cognition of depressive patients through the regulation of basal ganglia connectivity: combined medication using Shuganjieyu capsule. J Psychiatr Res. (2020) 123:39–47. 10.1016/j.jpsychires.2020.01.013 32035307

[8] Sarris J. Herbal medicines in the treatment of psychiatric disorders: 10-year updated review. Phytother Res. (2018) 32:1147–62. 10.1002/ptr.6055 29575228

[9] Ben-Eliezer D Yechiam E. *Hypericum perforatum* as a cognitive enhancer in rodents: a meta-analysis. Sci Rep. (2016) 6:35700. 10.1038/srep35700 27762349 PMC5071825

[10] Muszyńska B Łojewski M Rojowski J Opoka W Sułkowska-Ziaja K. Natural products of relevance in the prevention and supportive treatment of depression. Psychiatr Pol. (2015) 49:435–53. 10.12740/PP/29367 26276913

[11] Huang HJ Huang CY Lee M Lin JY Hsieh-Li HM. Puerariae radix prevents anxiety and cognitive deficits in mice under Oligomeric Aβ-Induced stress. Am J Chin Med. (2019) 47:1459–81. 10.1142/S0192415X19500757 31752523

[12] Ng QX Venkatanarayanan N Ho CY. Clinical use of *Hypericum perforatum* (St John's wort) in depression: a meta-analysis. J Affect Disord. (2017) 210:211–21. 10.1016/j.jad.2016.12.048 28064110

[13] Zhang M Bai X. Shugan jieyu capsule in post-stroke depression treatment: from molecules to systems. Front Pharmacol. (2022) 13:821270. 10.3389/fphar.2022.821270 35140618 PMC8818889

[14] Lee MH Smyser CD Shimony JS. Resting-state fMRI: a review of methods and clinical applications. AJNR Am J Neuroradiol. (2013) 34:1866–72. 10.3174/ajnr.A3263 22936095 PMC4035703

[15] Oestreich LKL Wright P O'Sullivan MJ. Microstructural changes in the reward system are associated with post-stroke depression. Neuroimage Clin. (2020) 28:102360. 10.1016/j.nicl.2020.102360 32795963 PMC7426585

[16] Jaywant A DelPonte L Kanellopoulos D O'Dell MW Gunning FM. The structural and functional neuroanatomy of post-stroke depression and executive dysfunction: a review of neuroimaging findings and implications for treatment. J Geriatr Psychiatry Neurol. (2022) 35:3–11. 10.1177/0891988720968270 33073704

[17] Yao G Li J Wang J Liu S Li X Cao X. Improved resting-state functional dynamics in post-stroke depressive patients after shugan jieyu capsule treatment. Front Neurosci. (2020) 14:297. 10.3389/fnins.2020.00297 32372901 PMC7177051

[18] Tang C Zhang Y Zhai Z Zhu X Wang C Yang G. Mechanism of Depression through brain function imaging of depression patients and normal people. J Healthc Eng. (2022) 2022:1125049. 10.1155/2022/1125049 35047144 PMC8763528

[19] Li G Zhang W Hu Y Wang J Li J Jia Z. Distinct basal brain functional activity and connectivity in the emotional-arousal network and thalamus in patients with functional constipation associated with anxiety and/or depressive disorders. Psychosom Med. (2021) 83:707–14. 10.1097/PSY.0000000000000958 34117157

[20] Ji L Meda SA Tamminga CA Clementz BA Keshavan MS Sweeney JA. Characterizing functional regional homogeneity (ReHo) as a B-SNIP psychosis biomarker using traditional and machine learning approaches. Schizophr Res. (2020) 215:430–8. 10.1016/j.schres.2019.07.015 31439419

[21] Zhao X Yao J Lv Y Zhang X Han C Chen L. Abnormalities of regional homogeneity and its correlation with clinical symptoms in Naïve patients with first-episode schizophrenia. Brain Imaging Behav. (2019) 13:503–13. 10.1007/s11682-018-9882-4 29736883

[22] Sun H Luo L Yuan X Zhang L He Y Yao S. Regional homogeneity and functional connectivity patterns in major depressive disorder, cognitive vulnerability to depression and healthy subjects. J Affect Disord. (2018) 235:229–35. 10.1016/j.jad.2018.04.061 29660636

[23] Zou QH Zhu CZ Yang Y Zuo XN Long XY Cao QJ. An improved approach to detection of amplitude of low-frequency fluctuation (ALFF) for resting-state fMRI: fractional ALFF. J Neurosci Methods. (2008) 172:137–41. 10.1016/j.jneumeth.2008.04.012 18501969 PMC3902859

[24] Liu CH Tang LR Gao Y Zhang GZ Li B Li M. Resting-state mapping of neural signatures of vulnerability to depression relapse. J Affect Disord. (2019) 250:371–9. 10.1016/j.jad.2019.03.022 30877860

[25] Kilts CD Wade AG Andersen HF Schlaepfer TE. Baseline severity of depression predicts antidepressant drug response relative to escitalopram. Expert Opin Pharmacother. (2009) 10:927–36. 10.1517/14656560902849258 19317630

[26] Brett M Leff AP Rorden C Ashburner J. Spatial normalization of brain images with focal lesions using cost function masking. Neuroimage. (2001) 14:486–500. 10.1006/nimg.2001.0845 11467921

[27] M W. Effect of Shugan Jieyu capsule on cognitive function and sleep quality in patients with post-stroke depression. China Sanit Stand Manag. (2016). 7:136–8. https://kns.cnki.net/kcms/detail/detail.aspx?dbcode=CJFD&dbname=CJFDLAST2016&filename=WSBZ201617098&uniplatform=NZKPT&v=ZXb1WZMVPKQ49HL2vT2eiq_0p_zEgt5uvzZqoJuUVOKaQw46a5R_cgOww5kDCbdV

[28] Balaev V Orlov I Petrushevsky A Martynova O. Functional connectivity between salience, default mode and frontoparietal networks in post-stroke depression. J Affect Disord. (2018) 227:554–62. 10.1016/j.jad.2017.11.044 29169125

[29] Zhang P Wang J Xu Q Song Z Dai J Wang J. Altered functional connectivity in post-ischemic stroke depression: a resting-state functional magnetic resonance imaging study. Eur J Radiol. (2018) 100:156–65. 10.1016/j.ejrad.2018.01.003 29373162

[30] Shi Y Zeng Y Wu L Liu W Liu Z Zhang S. A study of the brain abnormalities of post-stroke depression in frontal lobe lesion. Sci Rep. (2017) 7:13203. 10.1038/s41598-017-13681-w 29038494 PMC5643375

[31] Egorova N Cumming T Shirbin C Veldsman M Werden E Brodtmann A. Lower cognitive control network connectivity in stroke participants with depressive features. Transl Psychiatry. (2018) 7:4. 10.1038/s41398-017-0038-x 29520018 PMC5843603

[32] Wei HL Zhou X Chen YC Yu YS Guo X Zhou GP. Impaired intrinsic functional connectivity between the thalamus and visual cortex in migraine without aura. J Headache Pain. (2019) 20:116. 10.1186/s10194-019-1065-1 31856703 PMC6924083

[33] Yang M Li J Yao D Chen H. Disrupted intrinsic local synchronization in poststroke aphasia. Medicine. (2016) 95:e3101. 10.1097/MD.0000000000003101 26986152 PMC4839933

[34] Qiu S Chen F Chen G Jia Y Gong J Luo X. Abnormal resting-state regional homogeneity in unmedicated bipolar II disorder. J Affect Disord. (2019) 256:604–10. 10.1016/j.jad.2019.06.037 31299441

[35] Frank DW Dewitt M Hudgens-Haney M Schaeffer DJ Ball BH Schwarz NF. Emotion regulation: quantitative meta-analysis of functional activation and deactivation. Neurosci Biobehav Rev. (2014) 45:202–11. 10.1016/j.neubiorev.2014.06.010 24984244

[36] Cheng B Zhou Y Kwok VPY Li Y Wang S Zhao Y. Altered functional connectivity density and couplings in postpartum depression with and without anxiety. Soc Cogn Affect Neurosci. (2021) nsab127. 10.1093/scan/nsab127 34904174 PMC9340108

[37] Chen L Wang Y Niu C Zhong S Hu H Chen P. Common and distinct abnormal frontal-limbic system structural and functional patterns in patients with major depression and bipolar disorder. Neuroimage Clin. (2018) 20:42–50. 10.1016/j.nicl.2018.07.002 30069426 PMC6067086

[38] Zhang K Liu Z Cao X Yang C Xu Y Xu T. Amplitude of low-frequency fluctuations in first-episode, drug-naïve depressive patients: a 5-year retrospective study. PloS ONE. (2017) 12:e0174564. 10.1371/journal.pone.0174564 28384269 PMC5383053

[39] Wang S Zhao Y Zhang L Wang X Wang X Cheng B. Stress and the brain: perceived stress mediates the impact of the superior frontal gyrus spontaneous activity on depressive symptoms in late adolescence. Hum Brain Mapp. (2019) 40:4982–93. 10.1002/hbm.24752 31397949 PMC6865488

[40] Sereno MI Huang RS. Multisensory maps in parietal cortex. Curr Opin Neurobiol. (2014) 24:39–46. 10.1016/j.conb.2013.08.014 24492077 PMC3969294

[41] Liu C Han T Xu Z Liu J Zhang M Du J. Modulating gamma oscillations promotes brain connectivity to improve cognitive impairment. Cereb Cortex. (2021) bhab371. 10.1093/cercor/bhab371 34751749

[42] Schneider S Peters J Peth JM Büchel C. Parental inconsistency, impulsive choice and neural value representations in healthy adolescents. Transl Psychiatry. (2014) 4:e382. 10.1038/tp.2014.20 24736798 PMC4012284

[43] Power JD Petersen SE. Control-related systems in the human brain. Curr Opin Neurobiol. (2013) 23:223–8. 10.1016/j.conb.2012.12.009 23347645 PMC3632325

[44] Vicentini JE Weiler M Almeida SRM de Campos BM Valler L Li LM. Depression and anxiety symptoms are associated to disruption of default mode network in subacute ischemic stroke. Brain Imaging Behav. (2017) 11:1571–80. 10.1007/s11682-016-9605-7 27743373

[45] Wang J Xie S Guo X Becker B Fox PT Eickhoff SB. Correspondent functional topography of the human left inferior parietal lobule at rest and under task revealed using resting-State fMRI and coactivation based parcellation. Hum Brain Mapp. (2017) 38:1659–75. 10.1002/hbm.23488 28045222 PMC6867154

[46] Wang J Wei Q Wang L Zhang H Bai T Cheng L. Functional reorganization of intra- and internetwork connectivity in major depressive disorder after electroconvulsive therapy. Hum Brain Mapp. (2018) 39:1403–11. 10.1002/hbm.23928 29266749 PMC6866547

[47] Lassalle-Lagadec S Sibon I Dilharreguy B Renou P Fleury O Allard M. Subacute default mode network dysfunction in the prediction of post-stroke depression severity. Radiology. (2012) 264:218–24. 10.1148/radiol.12111718 22668562

